# pH Low Insertion Peptide-Modified Programmed Cell Death-Ligand 1 Potently Suppresses T-Cell Activation Under Acidic Condition

**DOI:** 10.3389/fimmu.2021.794226

**Published:** 2021-12-23

**Authors:** Ying Sun, Linhan Hu, Peng Yang, Min Zhang, Xinwei Wang, He Xiao, Chunxia Qiao, Jing Wang, Longlong Luo, Jiannan Feng, Yuanqiang Zheng, Yi Wang, Yanchun Shi, Guojiang Chen

**Affiliations:** ^1^ Inner Mongolia Key Lab of Molecular Biology, School of Basic Medical Sciences, Inner Mongolia Medical University, Hohhot, China; ^2^ State Key Laboratory of Toxicology and Medical Countermeasures, Institute of Pharmacology and Toxicology, Beijing, China; ^3^ Department of Hematology, The Fifth Medical Center, Chinese PLA General Hospital, Beijing, China

**Keywords:** PD-L1, pHLIP, inflammation, acidic, immunosuppression

## Abstract

Programmed cell death-ligand 1 (PD-L1)/PD-1 axis is critical for maintenance of immune homeostasis by limiting overactivation of effector T-cell responses. The impairment of PD-L1/PD-1 signals play an important role in the pathogenesis of inflammatory diseases, making this pathway an ideal target for novel therapeutics to induce immune tolerance. Given weakly acidic environment as a putative hallmark of inflammation, in this study we designed a new cargo by linking the ectodomain of murine PD-L1 to the N terminus of pHLIPs, a low pH-responding and membrane-insertion peptide, and demonstrated its potent immune-suppressive activity. Specifically, PD-L1-pHLIP spanned the cellular membrane and perfectly recognized its ligand PD-1 in acidic buffer. Immobile PD-L1-pHLIP actively inhibited T-cell proliferation and IFN-γ production. Importantly, soluble PD-L1-pHLIP retained its function to dampen T-cell responses under acidic condition instead of neutral aqueous solution. Overall, these data suggest that PD-L1-pHLIP has potentials to be a novel therapeutic avenue for T-cell-mediated inflammatory diseases.

## Introduction

Programmed cell death-ligand 1 (PD-L1) is the ligand for the inhibitory PD-1 receptor, the latter expressed primarily on activated T cells (1). Crosslinking of PD-L1 and PD-1 inhibits T-cell proliferation and cytokine production, which is critical for the induction and maintenance of immune tolerance (2, 3). Genetic deletion of PD-1 leads to the development of various autoimmune diseases depending on the stains (4–6). Similarly, PD-L1^−/−^ mice are more susceptible to experimental autoimmune encephalomyelitis, which is associated with the augmented responses of self-reactive CD4^+^ T cells *in vivo (* 7). In nonobese diabetic (NOD) mice, PD-1 or PD-L1 deficiency accelerates the onset of type 1 diabetes (3, 8). Of note, transgenic expression of PD-L1 in pancreatic β cells in NOD mice significantly decreases the severity of insulitis and delays disease onset as well as reduces the incidence of diabetes (9, 10), indicating that increased expression of PD-L1 in the lesions represents a potentially therapeutic strategy for autoimmune diseases.

Chronic inflammation is the hallmark of autoimmune diseases, characterized by massive infiltration and activation of immune cells (11, 12). Notably, enhanced glycolysis inherently occurs in lipopolysaccharide (LPS)-activated macrophages and dendritic cells (13, 14), in activated effector T cells, as well as other lymphocytes (15–17), which enables the immune cell to generate sufficient ATP and biosynthetic intermediates to carry out its particular effector functions (18, 19). Consequently, lactate, the well-known byproducts of glycolysis, accumulates in extracellular space, leading to acidosis (conventionally less than 7.0 of pH) at the site of inflammation (20–22). pHLIPs are a family of soluble ~36 amino acid peptides with pH-dependent transmembrane activity (23). When the environment is acidic, a pHLIP folds and inserts across the membrane to form a stable transmembrane helix, thus preferentially locating itself in acidic tissues (24). The low-pH targeting behavior of pHLIPs leads to applications as carriers for diagnostic and surgical imaging agents or delivery of cell-impermeable cargos into the cytosol of targeted cells (25, 26). In addition, a variety of other cargos, such as small molecule antigen (e.g. 2,4-dinitrophenyl (DNP)), can be associated with N terminus of pHLIPs and specifically targeted to the surfaces of cancer cells to induce biological responses, since tumors are putatively acidic (27). In this study, we engage the extracellular region of murine PD-L1 with the N terminus of pHLIPs and evaluate the function of this fusion protein (PD-L1-pHLIP) to suppress lymphocyte expansion and cytokine production in acidic buffer, providing a potentially innovative avenue to treat inflammatory autoimmune diseases.

## Materials and Methods

### Expression and Purification of Recombinant Proteins

The amino acid sequences of recombinant proteins used in this study were indicated as below. All constructs carried a C-terminal histidine tag or IgG1 Fc fragment for purification and were cloned into pMT-puro vectors for expression in HEK293 cells. Stably transfected cells were selected with 6 μg/ml puromycin. The resulting proteins were purified using Ni-affinity or protein A agarose affinity chromatography column (Cytiva, Amersham Place, UK) followed by further purification by SEC using a Superdex 200 (S200) column with 10 mM Tris and 150 mM NaCl, pH 7.5 (1× TBS). The purity of recombinant proteins was determined by SDS-PAGE (GenScript, Nanjing, China).

Protein sequences used in this study:

Murine PD-L1-WT pHLIP (wild-type pHLIP was used here, denoted to PD-L1-pHLIP):

MDAMKRGLCCVLLLCGAVFVSNSHHHHHHHHFTITAPKDLYVVEYGSNVTMECRFPVERELDLLALVVYWEKEDEQVIQFVAGEEDLKPQHSNFRGRASLPKDQLLKGNAALQITDVKLQDAGVYCCIISYGGADYKRITLKVNAPYRKINQRISVDPATSEHELICQAEGYPEAEVIWTNSDHQPVSGKRSVTTSRTEGMLek?>LNVTSSLRVNATANDVFYCTFWRSQPGQNHTAELIIPELPATHPPQNRTGGGGSGGGGSGGGGSAEQNPIYWARYADWLFTTPLLLLDLALLVDADEGT

Murine PD-L1-mFc (denoted to PD-L1-Fc):

MDAMKRGLCCVLLLCGAVFVSNSFTITAPKDLYVVEYGSNVTMECRFPVERELDLLALVVYWEKEDEQVIQFVAGEEDLKPQHSNFRGRASLPKDQLLKGNAALQITDVKLQDAGVYCCIISYGGADYKRITLKVNAPYRKINQRISVDPATSEHELICQAEGYPEAEVIWTNSDHQPVSGKRSVTTSRTEGMLLNVTSSLRVNATANDVFYCTFWRSQPGQNHTAELIIPELPATHPPQNRTDDDDKAVPRDSGCKPCICTVPEVSSVFIFPPKPKDVLTITLTPKVTCVVVDISKDDPEVQFSWFVDDVEVHTAQTQPREEQFNSTFRSVSELPIMHQDWLNGKEFKCRVNSAAFPAPIEKTISKTKGRPKAPQVYTIPPPKEQMAKDKVSLTCMITDFFPEDITVEWQWNGQPAENYKNTQPIMDTDGSYFVYSKLNVQKSNWEAGNTFTCSVLHEGLHNHHTEKSLSHSPGK

Murine PD-L1-mutated WT pHLIP (denoted to PD-L1-pHLIP(m)):

MDAMKRGLCCVLLLCGAVFVSNSHHHHHHHHFTITAPKDLYVVEYGSNVTMECRFPVERELDLLALVVYWEKEDEQVIQFVAGEEDLKPQHSNFRGRASLPKDQLLKGNAALQITDVKLQDAGVYCCIISYGGADYKRITLKVNAPYRKINQRISVDPATSEHELICQAEGYPEAEVIWTNSDHQPVSGKRSVTTSRTEGML1LNVTSSLRVNATANDVFYCTFWRSQPGQNHTAELIIPELPATHPPQNRTGGGGSGGGGSGGGGSACEQNPIYWARYAKWLFTTPLLLLKLALLVDADEGT

EBOV-secreted glycoprotein (sGP)-WT pHLIP (denoted to control protein):

MDAMKRGLCCVLLLCGAVFVSNSHHHHHHIPLGVIHNSTLQVSDVDKLVCRDKLSSTNQLRSVGLNLEGNGVATDVPSATKRWGFRSGVPPKVVNYEAGEWAENCYNLEIKKPDGSECLPAAPDGIRGFPRCRYVHKVSGTGPCAGDFAFHKEGAFFLYDRLASTVIYRGTTFAEGVVAFLILPQAKKDFFSSHPLREPVNATEDPSSGYYSTTIRYQATGFGTNETEYLFEVDNLTYVQLESRFTPQFLLQLNETIYASGKRSNTTGKLIWKVNPEIDTTIGEWAFWETKGGGGSGGGGSGGGGSACEQNPIYWARYADWLFTTPLLLLDLALLVDADEGT

### ELISA

A total of 96-well plated were coated overnight with the indicated antigens at 2 or 5 μg/ml, washed three times with PBS-T, and blocked with 4% nonfat dried milk in PBS for 1 h at room temperature. Twofold serial dilution of biotin-labeled anti-mPD-L1 antibody (BioLegend, San Diego, CA, USA) from 6 μg/ml was measured. For mPD-1, which was biotinylated according to manufacturer’s instructions (Genemore, Shanghai, China), it was added at 25, 50, and 100 μg/ml and incubated for 1 h at room temperature. The wells were washed and avidin-conjugated HRP (1:4,000) in 0.5% BSA was added and incubated for 1 h at room temperature. A TMB substrate kit (Invitrogen, Waltham, MA, USA) was used for detection at 450 nm. IFN-γ levels in the supernatants were determined by sandwich ELSA assays (BioLegend, CA, USA).

### Cell Culture and Transient Transfection

Cell lines (THP-1, Raji, Jurkat, and HEK293T) were purchased from ATCC and cultured in Dulbecco’s modified Eagle’s medium (DMEM) or RPMI 1640 medium (Gibco, Waltham, MA, USA) supplemented with 10% fetal bovine serum and penicillin/streptomycin in a humidified atmosphere of 5% CO_2_ at 37°C. HEK293T cells were transiently transfected with mPD-1 and mPD-L1 expression plasmids using a PEI transfection protocol (Promega, Madison, WI, USA), respectively. 48 h later, the expression of PD-L1 and PD-1 on the surface of cells was detected by flow cytometry.

### Fluorescence Labeling of Proteins and Confocal Microscopic Imaging

Recombinant proteins were labeled by fluorescence dye (PE or AlexaFlour488) with Conjugation Kit, according to manufacturer’s protocol (Abcam, Cambridge, UK). The HEK293T or HCT116 cell lines were placed in a laser confocal dish (NEST, Wuxi, China) and cultured overnight. AlexaFlour488-conjugated protein was added at 10 µg/ml and cultured at 37°C for 1 h. Cells were washed twice with PBS with corresponding pH, and then the fluorescence on the surface of cell membrane was observed under confocal laser scanning microscope (Zeiss LSM880, Jena, Germany).

### Lymphocyte Isolation and BrdU Cell Proliferation Assays

The splenocytes of BALB/c mice (purchased from Charles River, Beijing, China) were isolated by gradient centrifugation. Human PBMC were isolated from whole blood of healthy donors with signed written informed consents. The indicated proteins at titrated concentrations were coated in 96-well plates (Corning, Christiansburg, VA, USA) at 4°C overnight. Lymphocytes were added (5 × 10^5^ cells/well) with stimulation of anti-CD3 and CD28 antibody cocktail (10 and 5 µg/ml, respectively) for 72 h. BrdU cell proliferation assays were performed according to the manufacturer’s protocol (Cell Signaling, Danvers, MA, USA). In addition, lymphocytes were treated as described above with soluble proteins indicated in PBS with corresponding pH or containing lactic acid (10 or 20 mM). In some settings, neutralizing mAbs to murine PD-1 or PD-L1 (10 μg/ml, BioLegend, CA, USA) were added.

### Flow Cytometry

The cells were incubated with PE-labeled proteins as indicated at 0.001–10 µg/ml for 0.5–4 h in PBS with corresponding pH or containing lactic acid (10 or 20 mM). In some settings, cells were incubated with the indicated proteins at 10 µg/ml for 1 h in PBS (pH 7.4 and 6.3). PE-labeled mPD-1 was added and incubated for another 30 min. Cells were washed twice with PBS with corresponding pH, and then detected by flow cytometry.

For BrdU incorporation, mouse spleen lymphocytes were treated as described above. Six hours before the end of stimulation, BrdU solution (Sigma, Darmstadt, Germany) with the final concentration of 10 µM was added. Cells were washed and incubated with APC-antimouse CD4/CD8 antibody (BioLegend, CA, USA) at 4°C for 30 min. After washing, fixing, and permeabilizing (Invitrogen, MA, USA), cells were incubated with PE-antimouse BrdU antibody (BioLegend, CA, USA) in the dark at 4°C for another 30 min. The fluorescence intensity was measured by FACS Calibur II (BD Biosciences, CA, USA).

### Quantitative RT-PCR

Extracted total mRNA with TRIzol reagent, Single-strand cDNA was made from 1µg total RNA by reverse transcription (RT) using a TransScript II First-Strand cDNA Synthesis SuperMix (TransGen Biotech, Beijing, China). qPCR was conducted using SYBR Green qPCR mixture (GenStar, Beijing, China) through 50 cycles in a IQ5 Real-Time PCR Detection Systems (Bio-Rad, Hercules, CA, USA). The sequences of the primers used for qPCR were as follows: the forward primer of mouse IFN-γ is 5’-ACAGCAAGGCGAAAAAGGATG-3’ and the reverse primer is 5’-TGGTGGACCACTCGGATGA-3’. The forward primer of mouse GAPDH is 5’-CATCAAGAAGGTGGTGAAGC-3’ and the reverse primer is 5’-CCTGTTGCTGTAGCCGTATT-3’. Data were analyzed using the delta-delta Ct method.

### Statistical Analysis

Data are expressed as the means ± SD according to at least 3 independent experiments. Two-tailed Student’s *t*-tests were used to compare experimental and control groups. Statistical significance is defined as *p* < 0.05.

## Results

### Recombinant Murine PD-L1-pHLIP Perfectly Binds to Its Ligand PD-1

**Figure 1:**
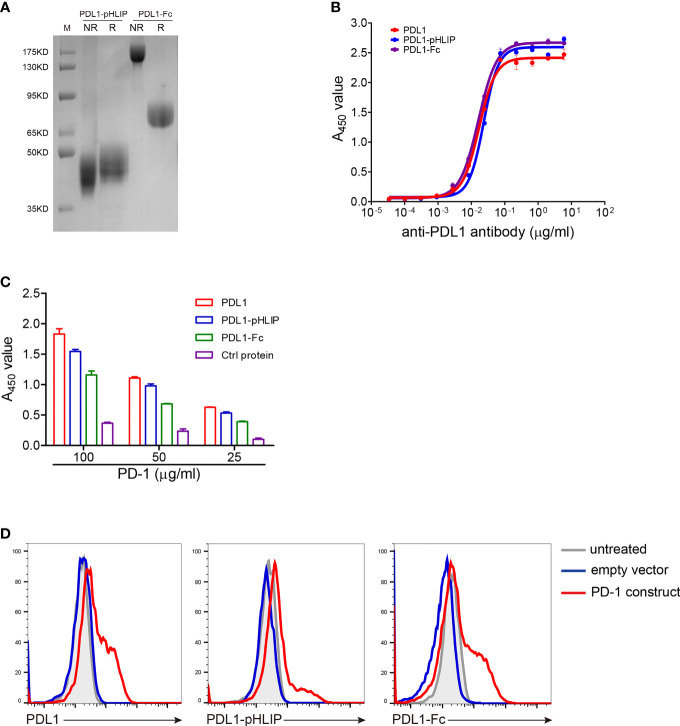
Validation of recombinant mouse PD-L1-pHLIP protein binding its ligand PD-1. **(A)** Recombinant PD-L1-pHLIP and PD-L1-Fc proteins were examined by SDS-PAGE. R, reducing; NR, nonreducing. **(B)** The binding capacity of PD-L1/PD-L1-pHLIP/PD-L1-Fc to an anti-PDL1 monoclonal antibody was determined by ELISA. **(C)** The ability of PD-L1/PD-L1-pHLIP/PD-L1-Fc to bind recombinant PD-1 protein was determined by ELISA. **(D)** Murine PD-1 construct was transiently introduced into HEK293T cell lines. The engagement of PE-conjugated PD-L1/PD-L1-pHLIP/PD-L1-Fc with membrane-bound PD-1 on the surface of HEK293T cells was examined by flow cytometry. Representative plots from three independent experiments were shown. The data were pooled from three experiments with similar results.

Firstly, the extracellular region of mouse PD-L1 was conjugated to the N terminus of wild-type pHLIPs using (G4S)_3_ as a coupling linker and the fusion protein generated in the eukaryotic expression system. As well, PD-L1 (having His tags) and PD-L1-Fc protein were also expressed using the same methods. The identities of these proteins were validated by SDS-PAGE (**Figure 1A**). Secondly, to determine whether pHLIP attachment affected native conformation of PD-L1, three experiments were performed as following: (1) The recognition of an antimurine PD-L1 antibody to these proteins was examined. The EC_50_ of PD-L1, PD-L1-pHLIP, and PD-L1-Fc was 0.017, 0.024, and 0.016 μg/ml, respectively (**Figure 1B**). This result indicated that these fusion proteins retained primary antigenic epitopes recognized by specific antibodies. (2) The binding capacity of these proteins to PD-1 was detected. Recombinant PD-1 protein (extracellular region) was expressed eukaryotically as described above (**Figure S1A**). To determine the binding ability of PD-1 protein to PD-L1, murine PD-L1 constructs were introduced into HEK293T cell line and its expression was confirmed by flow cytometry (**Figure S2A**). Importantly, fluorescence-labeled PD-1 protein was shown to recognize PD-L1-overexpressing HEK293T cells rather than empty vector-introduced controls (**Figure S1B**). Thereafter, the binding ability of these proteins to PD-1 was examined by ELISA. As shown in **Figure 1C**, the similar PD-1-bound capacity of PD-L1, PD-L1-pHLIP, and PD-L1-Fc was observed, suggesting that pHLIP conjugation did not influence the interaction between PD-L1 and PD-1. (3) The engagement of these proteins to membrane-bound PD-1 was determined. Murine PD-1 constructs were introduced into HEK293T cells, and its expression was validated (**Figure S2B**). The results showed that fluorescence-conjugated PD-L1, PD-L1-pHLIP, and PD-L1-Fc could recognize PD-1-knockin cells rather than empty vector-knockin controls (**Figure 1D**). Overall, these data indicate that pHLIP attachment has no significant effects on the engagement of PD-L1 fragment to PD-1.

### PD-L1-pHLIP Has Excellent Low pH-Responding Potentials to Insert the Membrane in Acidic Buffer

**Figure 2:**
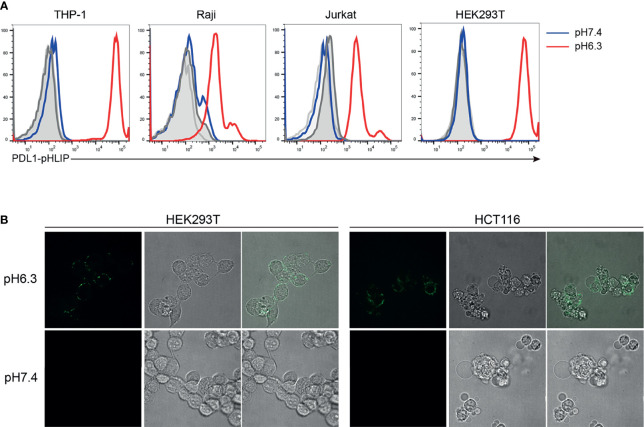
The low pH-responding ability of PD-L1-pHLIP to insert into cell membrane. **(A)** PE-conjugated PD-L1-pHLIP (10 μg/ml) was incubated with different cell lines indicated in pH 7.4 or 6.3 buffer for 1 h, respectively. The insertion ability was examined by flow cytometry. **(B)** AF488-conjugated PD-L1-pHLIP (10 μg/ml) was incubated with HEK293T or HCT116 cell lines in pH 7.4 or 6.3 buffer for 1 h, respectively. The insertion ability was examined by microscopic imaging. Representative images from three independent experiments were shown.

**Figure 3:**
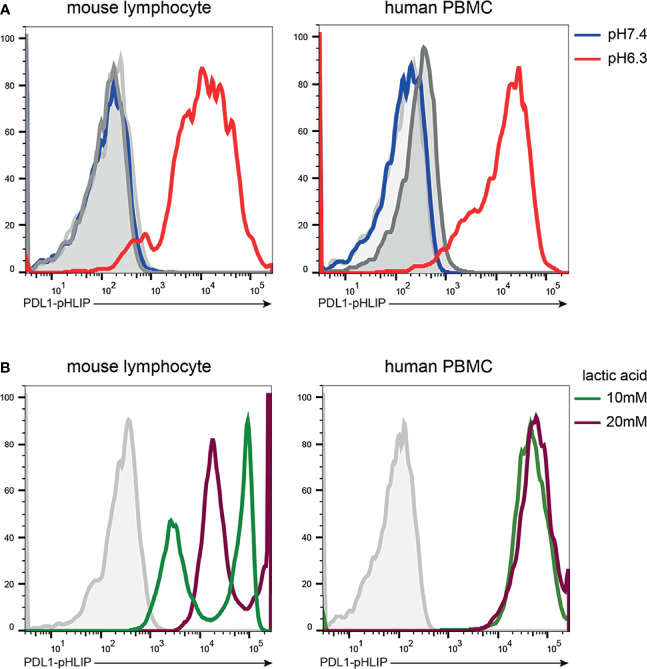
The low pH-responding ability of PD-L1-pHLIP to insert into primary murine and human lymphocytes. Mouse lymphocytes and human PBMC were isolated from the spleen of Balb/c strain and heathy volunteers, respectively. PE-conjugated PD-L1-pHLIP (10 μg/ml) were incubated with these cell populations for 1 h in pH 7.4 or 6.3 buffer **(A)** or buffer containing 10 or 20 mM lactic acid **(B)**. The fluorescence was examined by flow cytometry. Representative plots from three independent experiments were shown.

Whether PD-L1-pHLIP retains membrane-inserting capacity is an important issue for the implementation of its immune-suppressive function under acidic condition. To address this, several cell lines (HEK293T, THP-1, Raji, Jurkat) were incubated with fluorescence-labeled PD-L1-pHLIP for 1 h in pH 7.4 or 6.3 solutions and then detected by flow cytometry. No fluorescence was found in neutral aqueous solutions. In contrast, high magnitudes of fluorescence were visible in pH 6.3 buffer (**Figure 2A**), indicating that under acidic condition, PD-L1-pHLIP undergo conformational changes from random coil to α-helix and spans the cellular membrane. Moreover, fluorescent imaging also identified the exhibition of fluorescence-conjugated PD-L1-pHLIP on the cell surface in pH6.3 buffer instead of pH7.4 solutions (**Figure 2B**). Furthermore, we examined its membrane-inserting capacity in primary lymphocytes. As expected, PD-L1-pHLIP could potently spanned and displayed on the surface of mouse and human lymphocytes in pH6.3 buffer (**Figure 3A**). To accurately mimic acidic microenvironment *in vivo*, pH was titrated by lactic acid (10 or 20mM). Similar effects were observed (**Figure 3B**), which had important implications for the administration of PD-L1-pHLIP *in vivo*.

**Figure 4:**
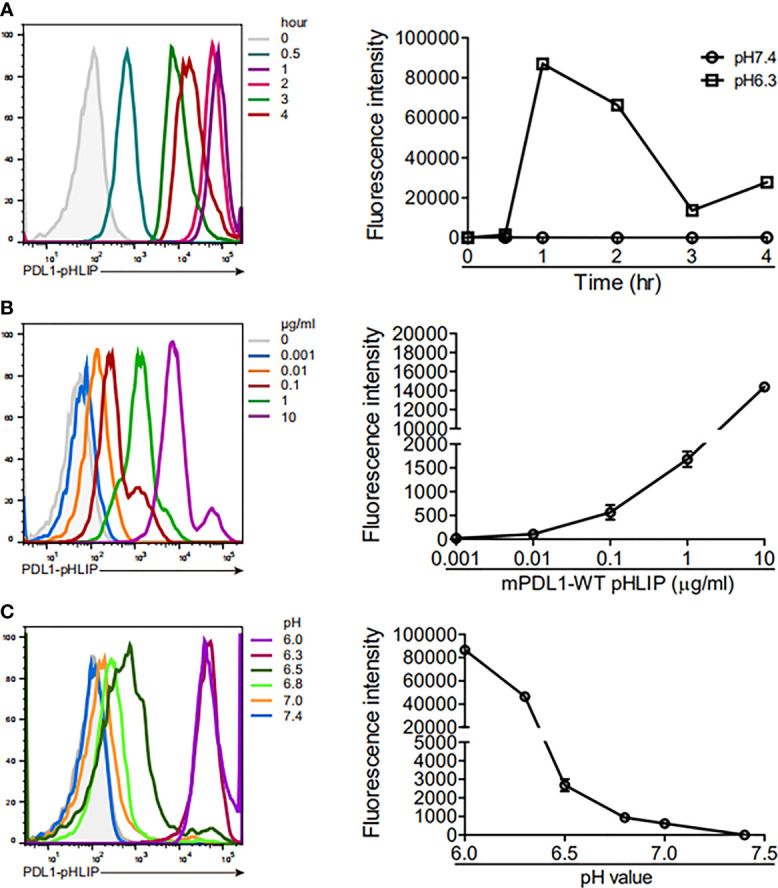
The ability of PD-L1-pHLIP to span across cell membrane under titrated conditions. **(A)** THP-1 cell lines were incubated with PE-conjugated PD-L1-pHLIP (10 μg/ml) for 0–4 h in pH 6.3 buffer and then examined by flow cytometry. **(B)** PE-conjugated PDL1-pHLIP (0–10 μg/ml) were incubated with THP-1 cell line for 1 h in pH 6.3 buffer and then examined by flow cytometry. **(C)** THP-1 cell lines were incubated with PE-conjugated PD-L1-pHLIP (10 μg/ml) for 1 h in pH 6.0–7.4 buffer and then examined by flow cytometry. Left, representative plots from three independent experiments. Right, the data of fluorescence intensity pooled.

Thereafter, we determined the membrane-spanning ability of PD-L1-pHLIP in the intervals of 0-4 hours under neutral or acidic conditions. PD-L1-pHLIP did not display membrane-inserting potentials in pH7.4 buffer, even for longer incubation time (**Figure S3**). Conversely, in acidic buffer, fluorescence intensity reach the peak at 1 hour after incubation, and then decreased over time. At 4-hour timepoint, the intensity remained relatively high (**Figure 4A**). Furthermore, we determined whether PD-L1-pHLIP insertion was dose and pH dependent. Indeed, fluorescence intensity of PD-L1-pHLIP elevated dramatically with the increase of concentration (**Figure 4B**). In a range of pH 6.0–7.4, the fluorescence intensity increased when pH of the solution was titrated (**Figure 4C**). Notably, significant elevation of the intensity was seen when pH value jumped down from 6.5 to 6.3 (**Figure 4C**), which might be associated with conformational transformation of PD-L1-pHLIP from the mixture of membrane-absorbed and membrane-inserted states to fully membrane-inserted states (28).

Next, we asked whether PD-L1-pHLIP displayed on the surface of cells could recognize PD-1 smoothly. PD-1 protein was labeled with fluorescent dye and added to THP-1 cell lines, which were preincubated with PD-L1-pHLIP in pH 7.4 or 6.3 buffer. No significant fluorescence was seen in neutral solution. In contrast, fluorescence was obvious in pH 6.3 buffer (**Figure S4A**). We also examined the engagement of fluorescence-conjugated PD-1 to other proteins (PD-L1, PD-L1-Fc, mutated PD-L1-pHLIP [named PD-L1-pHLIP(m)], control protein (EBOV sGP-pHLIP) under the same condition. No fluorescence was found (**Figure S4B**). Taken together, these data clearly indicate that PD-L1 attachment does not impair the acid-responding and membrane-spanning ability of pHLIPs and that its conformational changes have no or minimal effects on the interaction between PD-L1 and PD-1.

### Immobile PD-L1-pHLIP Potently Inhibits Lymphocyte Expansion and IFN-γ Secretion

We next determine the immune-suppressive activity of plate-coated PD-L1-pHLIP. As well known, PD-L1 delivers signals to T cells *via* binding to its ligands PD-1 or CD80 (29). PD-L1/PD-1 interaction is responsible for executing the immune-suppressive function of PD-L1. The expression levels of PD-1 in CD4^+^ and CD8^+^ T cells at different timepoints following TCR stimulation were detected respectively. In terms of CD4^+^ T cells, PD-1 levels were significantly elevated at 24 h post-treatment with the cocktail of anti-CD3/CD28 mAbs. Its expression was further augmented at 48 and 72 h (**Figure S5**). The distinct expression patterns of PD-1 were observed in CD8^+^ T cells. PD-1 levels in CD8^+^ T cells at 24 h were comparable with the resting cells. Its expression, however, increased drastically at 48 and 72 h following TCR stimulation (**Figure S5**).

**Figure 5:**
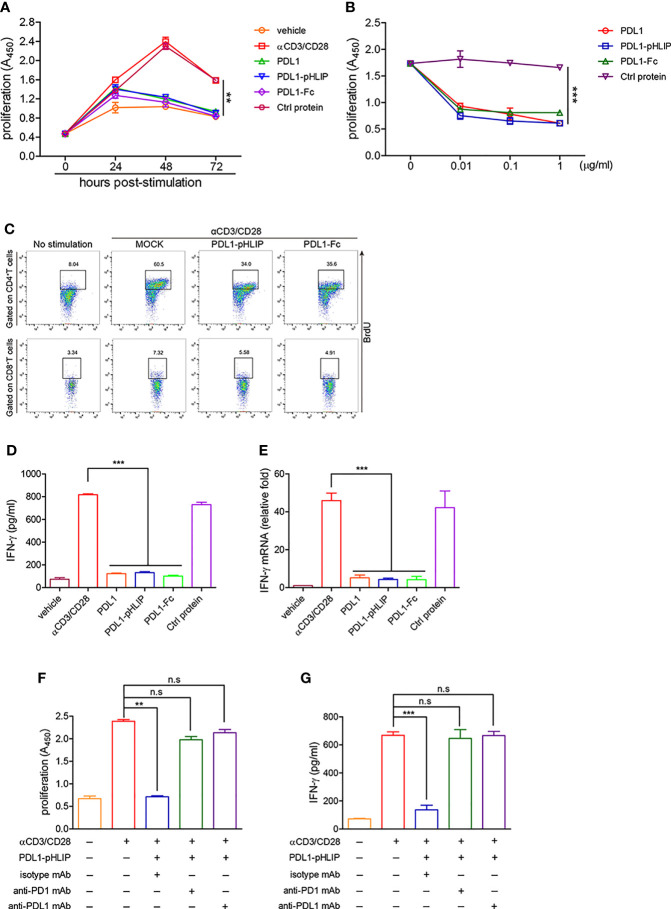
The capacity of immobile PD-L1-pHLIP to inhibit lymphocyte proliferation and IFN-γ production. **(A)** PD-L1/PD-L1-pHLIP/PD-L1-Fc or control protein (0.1 μg/ml) were coated in culture plates overnight. Mouse lymphocytes were isolated and stimulated with αCD3/CD28 antibodies for 0–72 h. The proliferation was determined by BrdU cell proliferation assays. **(B)** Mouse lymphocytes were stimulated with αCD3/CD28 antibodies in the absence or presence of immobile PD-L1/PD-L1-pHLIP/PD-L1-Fc (0–1 μg/ml) for 72 h. The proliferation was determined by BrdU cell proliferation assays. **(C)** Mouse lymphocytes were stimulated with αCD3/CD28 antibodies in the presence of immobile PD-L1/PD-L1-pHLIP/PD-L1-Fc (0.1 μg/ml) for 72 h. BrdU (10 μM) was incorporated at last 6 h. The proliferation of CD4^+^ or CD8^+^T lymphocytes was determined by flow cytometry. **(D, E)** Mouse lymphocytes were stimulated with αCD3/CD28 antibodies in the presence of immobile PD-L1/PD-L1-pHLIP/PD-L1-Fc (0.1 μg/ml) for 72 h. IFN-γ expression was examined by ELISA **(D)** and quantitative RT-PCR **(E)**, respectively. **(F, G)** Mouse lymphocytes were stimulated with αCD3/CD28 antibodies in the presence of immobile PD-L1/PD-L1-pHLIP/PD-L1-Fc (0.1 μg/ml) for 72 h. Monoclonal antibodies to PD-1 or PD-L1 or isotypes (10 μg/ml) were added in the culture. The proliferation was determined by BrdU cell proliferation assays **(F)**. IFN-γ production in the supernatants was examined by ELISA **(G)**. Representative plots from three independent experiments were shown. The data were pooled from five independent experiments with similar results. ^**^ *p* < 0.01; ^***^ *p* < 0.001; n.s., no significance.

Thereafter, we evaluated the function of plated-bound proteins (PD-L1-pHLIP, PD-L1, PD-L1-Fc, control protein) to inhibit proliferation of lymphocytes at the intervals of 24–72 h following treatment with anti-CD3/CD28 mAbs. At 24 h poststimulation, none of them (i.e., PD-L1-pHLIP, PD-L1, PD-L1-Fc) displayed inhibitory function. All of them, however, actively suppressed lymphocyte expansion at 48 and 72 h following TCR stimulation, with over 90% of inhibition rates (**Figure 5A**). Notably, control protein-conjugated pHLIPs did not exhibit proliferation-inhibitory capacity (**Figure 5A**). We further determine the dose-dependent effects of these proteins in a range of 0.01–1 μg/ml. The result showed that immobile proteins (PD-L1-pHLIP, PD-L1, PD-L1-Fc) still retained the strong potency of inhibition of lymphocyte proliferation at low concentration (0.01 μg/ml) (**Figure 5B**). To clarify the effects of these proteins on T-cell subsets, BrdU was incorporated into lymphocytes, followed by the detection of the frequencies of BrdU^+^ cells in CD4^+^ and CD8^+^ T-cell populations, respectively. These proteins (PD-L1-pHLIP, PD-L1-Fc), indeed, potently inhibited the expansion of CD4^+^ and CD8^+^ T cells simultaneously (**Figure 5C**). As well, immobile proteins were able to repress IFN-γ production by activated lymphocytes at the protein and mRNA levels (**Figures 5D, E** **)**. To determine whether PD-L1/PD-1 ligation was responsible for the inhibitory function of PD-L1-pHLIP, neutralizing antibodies to PD-L1 and PD-1 were added. Blocking PD-L1/PD-1 interaction using anti-PD-L1 or PD-1 antibody almost entirely abrogated the inhibitory ability of PD-L1-pHLIP, including suppressing lymphocyte proliferation and IFN-γ secretion (**Figures 5F, G** **)**. Overall, these results indicate that plate-bound PD-L1-pHLIP actively inhibits T lymphocyte expansion and cytokine production *via* binding to PD-1 receptor on the surface of T cells.

### PD-L1-pHLIP Exhibits Inhibitory Effects on Lymphocyte Proliferation and IFN-γ Production in Acidic Buffer

**Figure 6:**
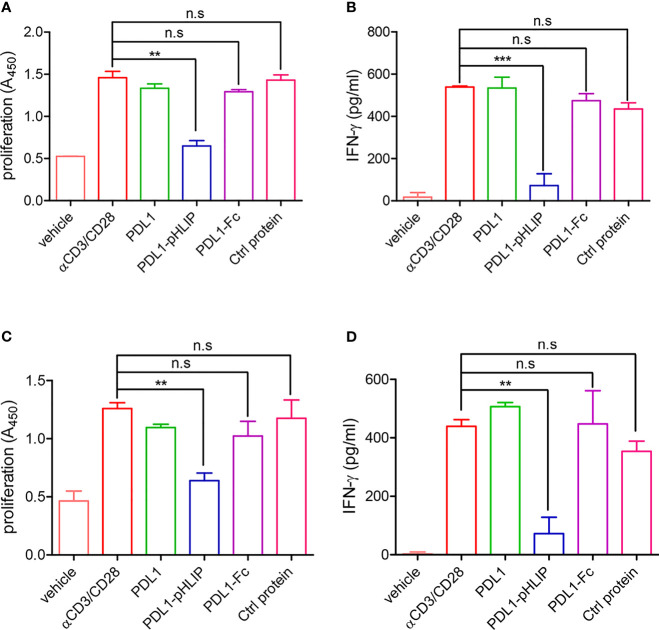
The capacity of soluble PD-L1-pHLIP to inhibit lymphocyte proliferation and IFN-γ production under acidic conditions. **(A, B)** Mouse lymphocytes were stimulated with αCD3/CD28 antibodies in the presence of soluble PD-L1/PD-L1-pHLIP/PD-L1-Fc (0.1 μg/ml) for 72 h in pH 6.3 buffer. The proliferation was determined by BrdU cell proliferation assays **(A)**. IFN-γ production in the supernatants was examined by ELISA **(B)**. **(C, D)** Mouse lymphocytes were stimulated with αCD3/CD28 antibodies in the presence of soluble PD-L1/PD-L1-pHLIP/PD-L1-Fc (0.1 μg/ml) for 72 h in buffer containing 10 mM lactic acid. The proliferation was determined by BrdU cell proliferation assays **(C)**. IFN-γ production in the supernatants was examined by ELISA **(D)**. The data were pooled from five independent experiments with similar results. ^**^ *p* < 0.01; ^***^ *p* < 0.001; n.s., no significance.

To test our hypothesis that under acidic conditions, PD-L1-pHLIP inserts and spans cellular membrane through conformational changes from unstructured coil to α helix, thereby playing an immune-suppressive role in T-cell activation *via* the interaction between PD-L1 and PD-1, we determined the inhibitory function of these proteins (PD-L1-pHLIP, PD-L1, PD-L1-Fc) at the soluble state in pH6.3 or 7.4 buffer respectively. As expected, these proteins at the soluble state did not suppress lymphocyte proliferation and IFN-γ secretion in neutral aqueous solution (**Figures S6A, B**). Of importance, in acidic buffer, soluble PD-L1-pHLIP robustly repressed lymphocyte expansion and IFN-γ production (**Figures 6A, B** **)**. In contrast, PD-L1 and PD-L1-Fc had no inhibitory effects on lymphocyte activation under the same condition (**Figures 6A, B** **)**. We further evaluated the function of these proteins in the solution containing 10mM lactic acid. Similarly, PD-L1-pHLIP displayed significantly inhibitory function on lymphocyte proliferation and IFN-γ production, instead of PD-L1 and PD-L1-Fc (**Figures 6C, D** **)**. Taken together, these data suggest that PD-L1-pHLIP actively suppresses T-cell activation under acidic condition.

## Discussion

The therapy for autoimmune diseases currently focus on the development of antagonists to block effector functions of one cytokine (e.g., IL-6, TNF-α, IL-17A, etc.) (30, 31). However, the onset of autoimmune diseases is well-known to result from the orchestrated effects of a variety of cytokines (32). In this study, we provide the proof of concept that PD-L1-pHLIP is designed to down-regulate the effector function of autoreactive T cells in the sites of inflammation, which is considered acidic. Given whole suppression of effector T-cell response instead of targeting one inflammatory mediators, PD-L1-pHLIP theoretically might be more effective to treat autoimmune diseases than targeting one cytokine, which need validation *in vivo* in the future. The reasons for choosing PD-L1 as a cargo are following: (a) PD-L1/PD-1 interaction is definitely critical to maintain T-cell tolerance and immune homeostasis. Long-term administration of PD-1 antibodies led to significant increase of risks for autoimmune diseases (e.g., type 1 diabetes), which was due to that PD-1 mAb treatment abrogated endogenous signaling axis of PD-L1/PD-1, thereby pathogenically activated autoreactive T cells and ultimately caused the onset of autoimmune diseases (33–35); (b) PD-1 expression is limited to activated T and B cells as well as myeloid cells, implying that PD-L1/PD-1 signals is an important brake to avoid excessive immune response. Since this signaling axis has minimal effects on the priming of immune reaction, augmenting PD-L1/PD-1 signals may have potentials to normalize immune responses (36); (c) PD-L1 is widely expressed in nonlymphoid tissues as well as on dendritic cells, allowing for regulation of potentially autoreactive lymphocytes at sites of immune activation as well as at effector sites. This may be a particularly important mechanism in limiting activities of both T and B cells in multiple organs where PD-L1 is highly expressed (37, 38); (d) Since that excessive inflammatory responses is partly ascribed to dampened PD-L1/PD-1 signals (39), we speculate that the increase of PD-L1 abundance and restoration of signaling strength in the site of inflammation can alleviate inflammatory reaction and tissue damage; (e) PD-L1 is type 1 membrane protein interacting with PD-1 at the state of monomers, which enables perfect recognition of the dissociated extracellular region of PD-L1 to membrane-bound PD-1 (40, 41); (f) The dissociated ectodomain of PD-L1 functions to antagonize PD-L1/PD-1 engagement. Upon anchoring the membrane by pHLIP-mediated insertion, pHLIP-modified PD-L1 plays an immune-suppressive role as full-length PD-L1.

Of note, there are some studies suggesting that soluble PD-L1 can inhibit T-cell activity *in vitro (* 42–44). Others suggest that this form of PD-L1 cannot do this but acts as antagonist to block the engagement of naïve PD-L1 with PD-1 on the surface of T cells (45–47). It is worthy to note that plate-coated PD-L1 protein can actively inhibit T-cell response (48–50). In addition, PD-L1-Fc administration exhibited therapeutic efficacy in several animal models of inflammatory diseases (48, 51, 52). This discrepancy can be resolved by the fact that crosslinking or aggregated IgG is required for activation of NK or T cells *via* FcγRIIA or FcγRIII (53, 54). Consequently, it is conceivable to assume that crosslinking of PD-L1 to PD-1 is necessary for inhibition of T-cell activity *in vitro*. Although soluble PD-L1 can bind to PD-1 *in vitro*, this form has no crosslinking activity so that it cannot elicit PD-1-mediated immune-suppressive response. The therapeutic effects of PD-L1-Fc *in vivo* may be attributed to Fc-mediated crosslinking of PD-L1 and PD-1 *via* interaction with Fc receptors on immune cells.

A recent study demonstrated that in NOD mice hematopoietic stem progenitor cells (HSPCs) were deficient in PD-L1 and transfusion of genetically engineered or pharmacologically modulated HSPCs overexpressing PD-L1 inhibited autoimmune response and reverted diabetes (9). However, given persistent overexpression of PD-L1 *in vivo*, this regimen could result in widespread immune suppression leading to increased risks for infection and cancer. Relatively, if our strategy is validated to be efficacious *in vivo*, the nonspecific immune suppression can be avoided and genetic manipulation can be prevented. The therapeutic efficacy might be largely affected by pH. When pH value dropped from 6.5 to 6.3 or even lower, the membrane-inserting ability of PD-L1-pHLIP improved significantly with increased membrane-bound abundances. This can be interpreted by the fact that at pH 6.4 pHLIPs exist as a mixture of a largely unstructured population (state II) and an inserted α-helical population (state III) (28). In lower pH (<6.4) buffer, pHLIPs exist at more stable membrane-inserted states thereby are anchored tightly on the surface of cells. In addition, the different pH in various diseases and dynamic alteration of pH at different stages of one disease may have an effect on membrane insertion and accumulation of pHLIPs in the lesion, which might greatly influence the therapeutic efficacy of PD-L1-pHLIP. This issue can be resolved in part by crosslinking pHLIP variants since some variants have ability of enhanced targeting and greater retention in acidic tissues in comparison with wild-type pHLIPs. In conclusions, our study have demonstrated that the extracellular region of PD-L1 tethering pHLIPs exhibits potently immune-suppressive activity and provide a potentially novel regimen for the treatment of autoimmune diseases and other T-cell-mediated inflammatory diseases.

## Data Availability Statement

The original contributions presented in the study are included in the article/**Supplementary Material**. Further inquiries can be directed to the corresponding authors.

## Ethics Statement

The studies involving human participants were reviewed and approved by The Ethics Committee of the Chinese PLA General Hospital. The patients/participants provided their written informed consent to participate in this study. The animal study was reviewed and approved by The Ethics Committee of the Academy of Beijing Institute of Pharmacology and Toxicology.

## Author Contributions

YiS and LH performed the experiments and prepared the manuscript. PY, MZ, and XW were involved in optimization of the experimental protocols. HX, CQ, JW, LL, JF, and YZ provided methodological support. YW, YaS, and GC conceived and guided the study. All authors contributed to the article and approved the submitted version.

## Funding

This work is supported by the National Natural Science Foundation of China (81672803, 81871252).

## Conflict of Interest

The authors declare that the research was conducted in the absence of any commercial or financial relationships that could be construed as a potential conflict of interest.

## Publisher’s Note

All claims expressed in this article are solely those of the authors and do not necessarily represent those of their affiliated organizations, or those of the publisher, the editors and the reviewers. Any product that may be evaluated in this article, or claim that may be made by its manufacturer, is not guaranteed or endorsed by the publisher.
