# Supplementary material for: pH Low Insertion Peptide-Modified Programmed Cell Death-Ligand 1 Potently Suppresses T-Cell Activation Under Acidic Condition
Source: Front Immunol. 2021 Dec 23;12:794226. doi: 10.3389/fimmu.2021.794226 (PMC8733706; doi:10.3389/fimmu.2021.794226)

**Supplementary figure legends**

**Figure S1. Validation of recombinant mouse PD-1 protein.** (A) Recombinant mouse PD-1 protein was examined by SDS-PAGE.(B) Murine PD-L1 construct was transiently introduced into HEK293T cell lines. The engagement of PE-conjugated PD-1 protein with membrane-bound PDL1 on the surface of HEK293T cells was examined by flow cytometry. Representative plots from two independent experiments were shown.

**Figure S2. Validation of murine PD-1 and PD-L1 expression in HEK293T cells.** Murine PDL1 and PD-1 constructs were transiently introduced into HEK293T cell line. 48h later, PD-L1 (A) and PD-1 (B) expression on the surface of cell membrane was examined by flow cytometry respectively. Representative plots from three independent experiments were shown.

**Figure S3. The inability of PD-L1-pHLIP to insert into cell membrane under pH7.4 condition.** PE-conjugated PD-L1-pHLIP (10μg/ml) were incubated with THP-1 cell line for 0-4h in pH7.4 buffer and then examined by flow cytometry. Representative plots from two independent experiments were shown.

**Figure S4. The ability of membrane-inserting PD-L1-pHLIP to recognize recombinant PD-1 protein.** (A) PD-L1-pHLIP (10μg/ml) was incubated with THP-1 cell line in pH7.4 or 6.3 buffer for 1h respectively. PE-conjugated PD-1 (1μg/ml) was then incubated for 30min. The insertion ability was examined by flow cytometry. (B) THP-1 cell line was incubated with recombinant murine PD-L1/PD-L1-Fc/PD-L1-pHLIP(m) or control protein (10μg/ml) in pH6.3 buffer for 1h respectively. PE-conjugated PD-1 (1μg/ml) was then incubated for 30min. The insertion ability was examined by flow cytometry. Representative plots from two independent experiments were shown.

**Figure S5. PD-1 expression on the surface of CD4^+^ and CD8^+^T cells.** Mouse lymphocytes were isolated from the spleen of Balb/c strain and stimulated with αCD3/CD28 antibodies for 0-72h. CD4^+^T and CD8^+^T cells were gated respectively and PD-1 expression in these subsets were examined by flow cytometry. Representative plots from two independent experiments were shown.

**Figure S6. The inability of soluble PD-L1-pHLIP to inhibit lymphocyte proliferation and IFN-γ production in pH7.4 buffer.** Mouse lymphocytes were stimulated with αCD3/CD28 antibodies in presence of soluble PD-L1/PD-L1-pHLIP/PD-L1-Fc (0.1μg/ml) for 72h in pH7.4 buffer. (A) The proliferation was determined by BrdU cell proliferation assays. (B) IFN-γ production in the supernatants was examined by ELISA. The data were pooled from three independent experiments with similar results. n.s, no significance.

Figure S1


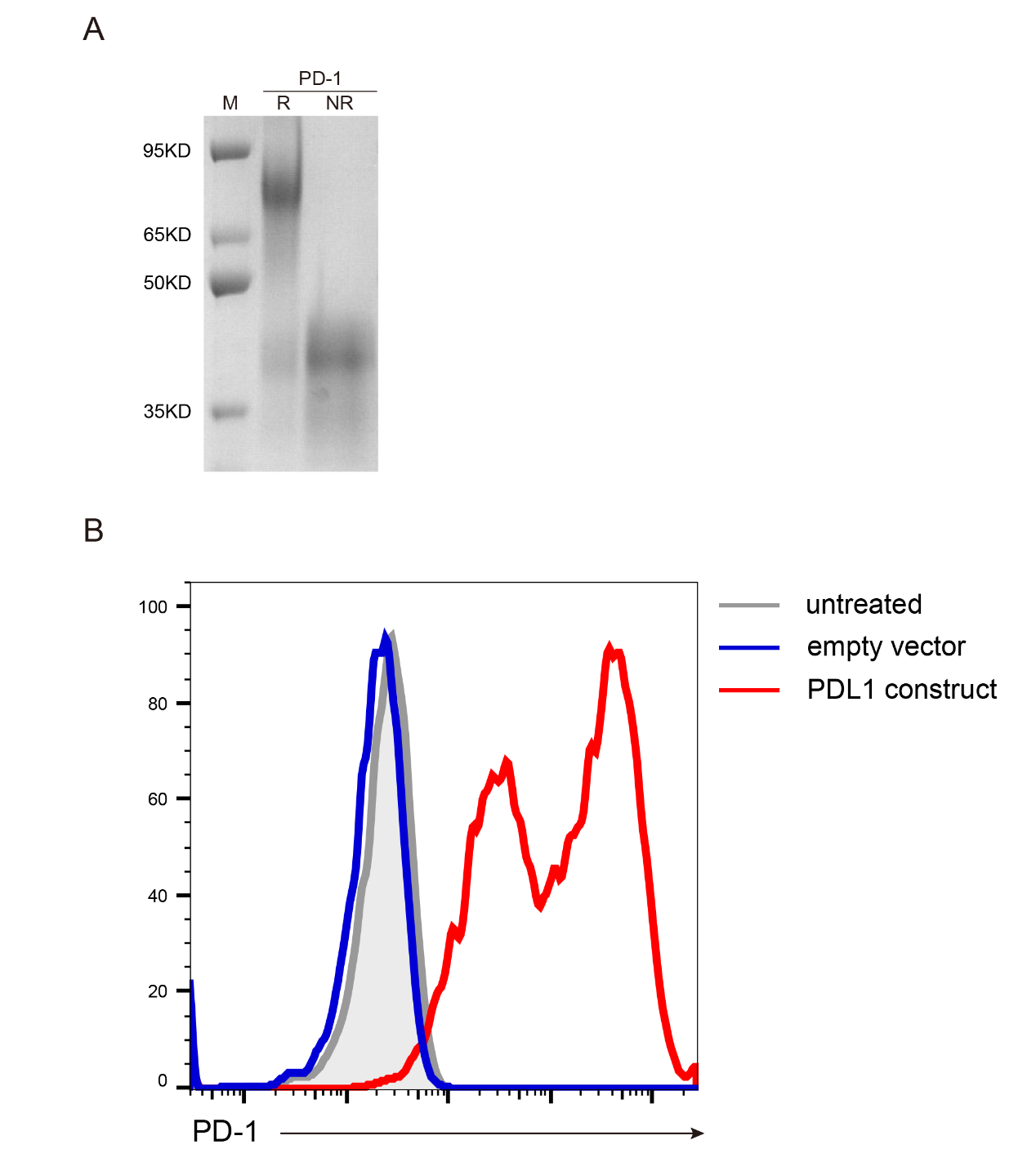


Figure S2


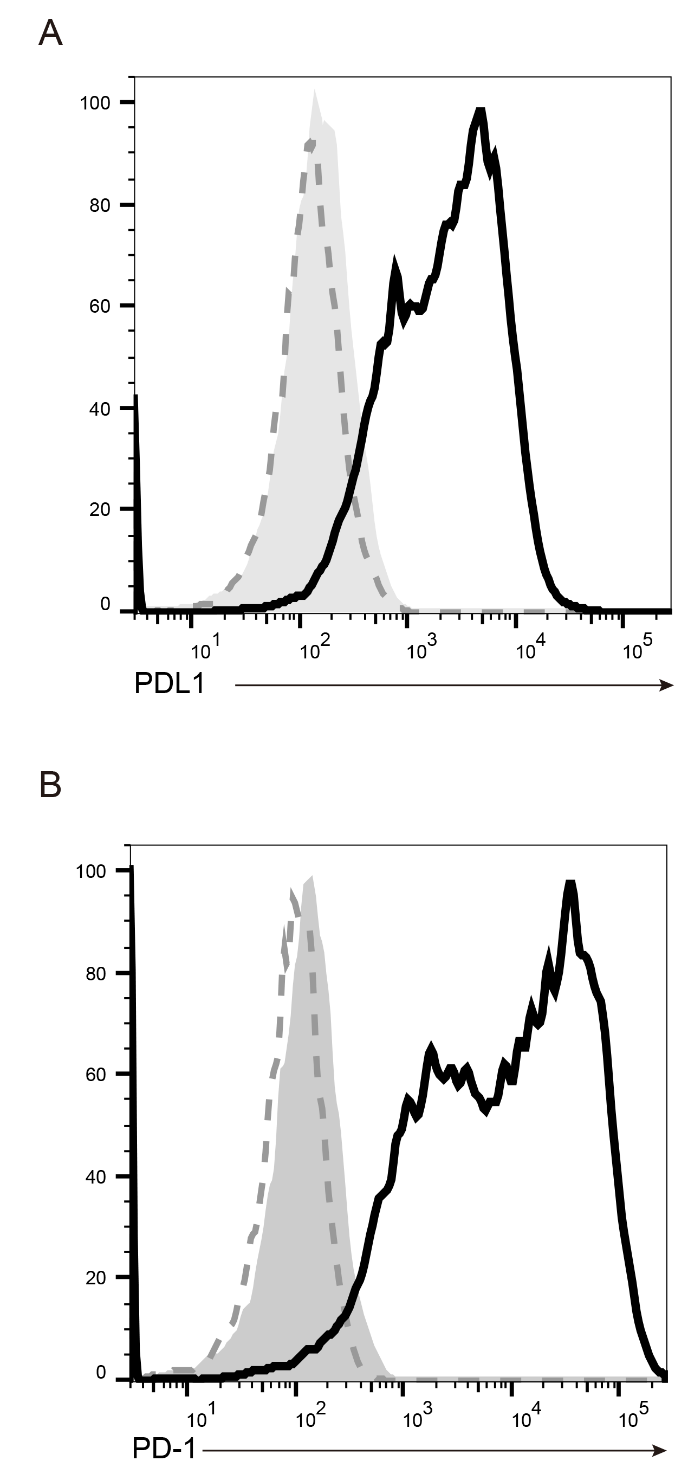


Figure S3


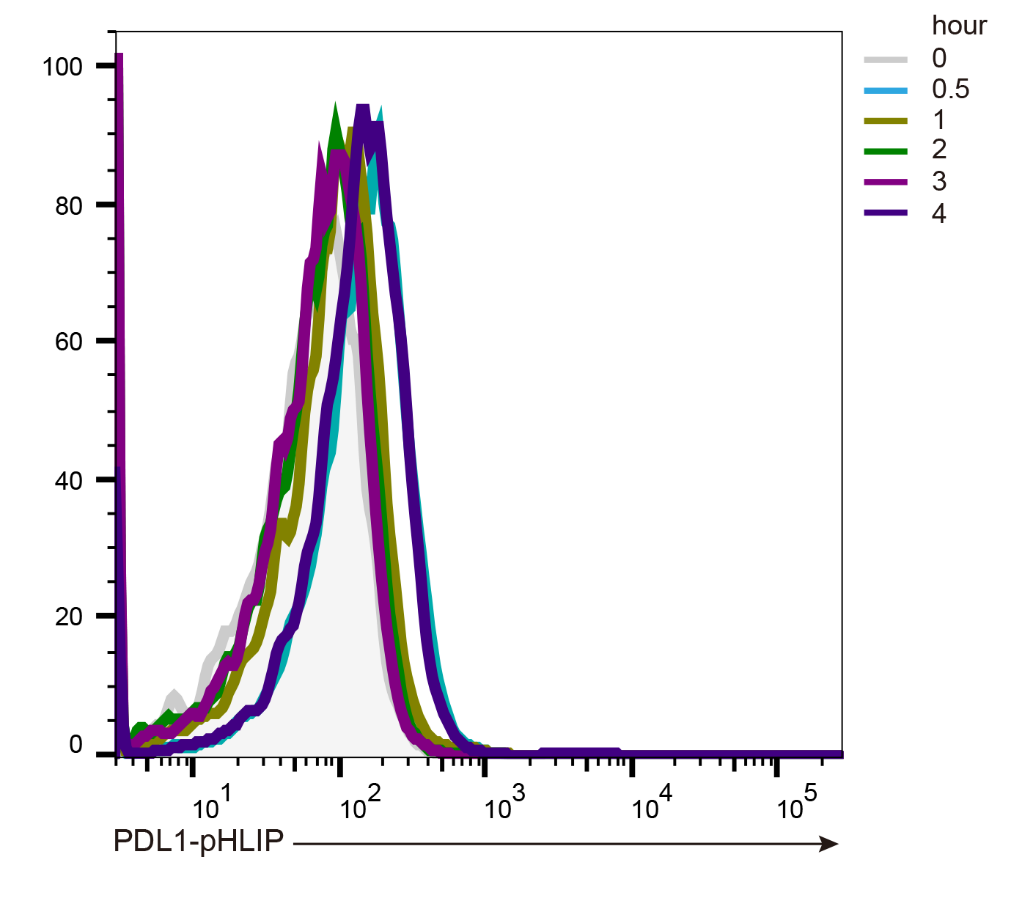


Figure S4


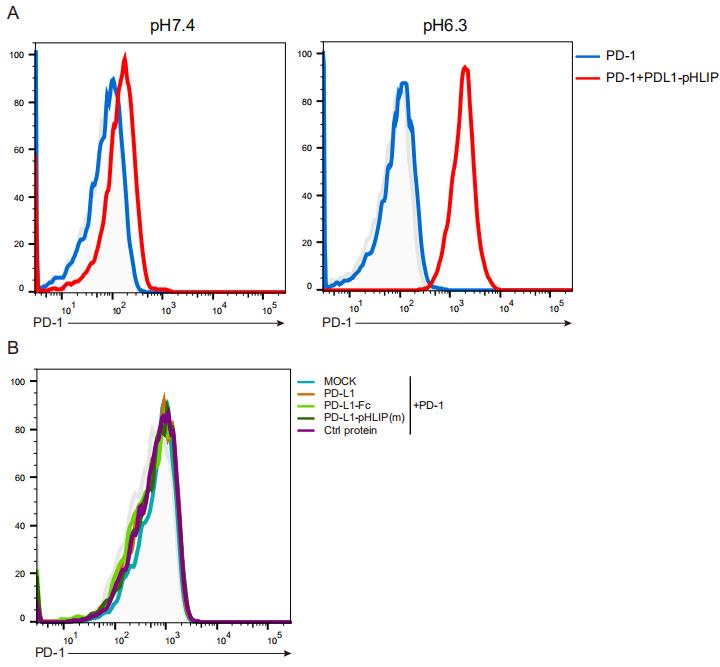


Figure S5


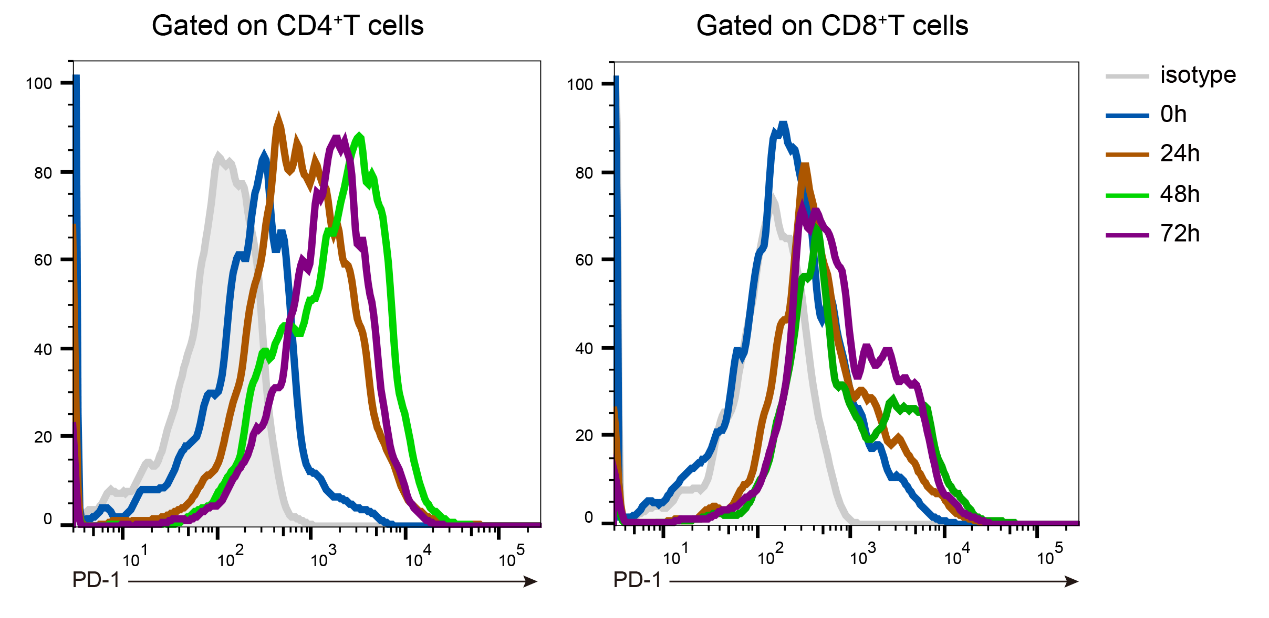


Figure S6


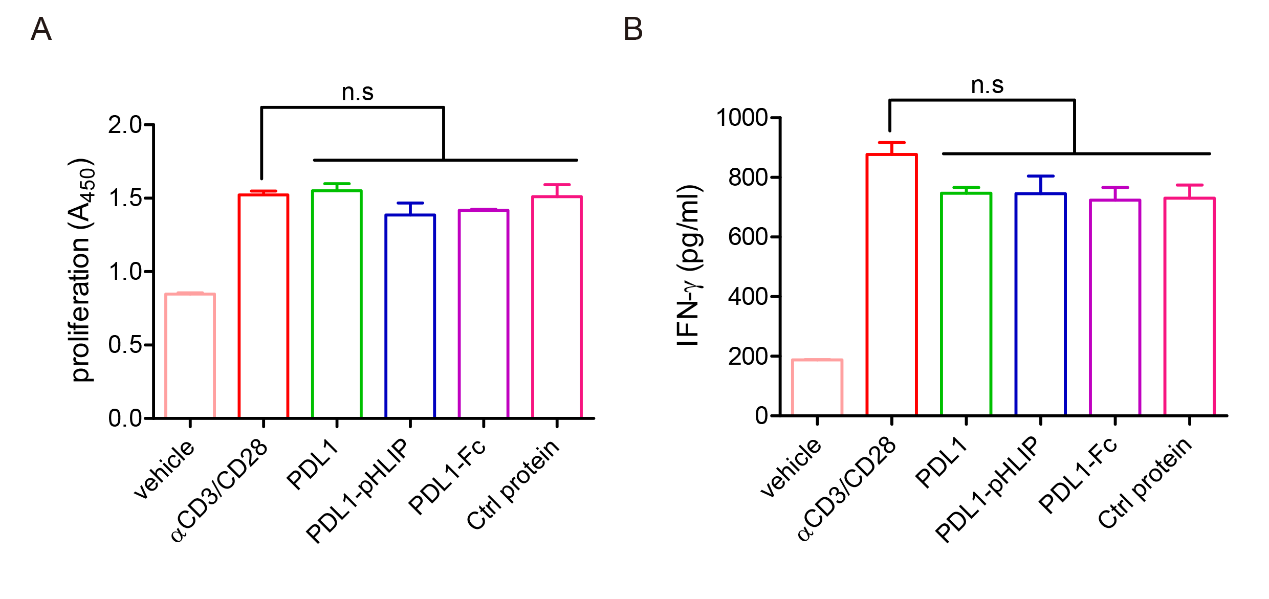

Supplement: Supplementary file 1 [file DataSheet_1.docx]
